# Implications of the genomic revolution for education research and policy

**DOI:** 10.1002/berj.3784

**Published:** 2022-04-12

**Authors:** Tim T. Morris, Stephanie von Hinke, Lindsey Pike, Neil R. Ingram, George Davey Smith, Marcus R. Munafò, Neil M. Davies

**Affiliations:** ^1^ Medical Research Council Integrative Epidemiology Unit University of Bristol Bristol UK; ^2^ Population Health Sciences Bristol Medical School University of Bristol Oakfield Grove Barley House Bristol UK; ^3^ School of Economics University of Bristol UK; ^4^ Erasmus School of Economics Erasmus University Rotterdam Rotterdam The Netherlands; ^5^ School of Education University of Bristol Bristol UK; ^6^ School of Psychological Science University of Bristol Bristol UK; ^7^ K.G. Jebsen Center for Genetic Epidemiology Department of Public Health and Nursing Norwegian University of Science and Technology Trondheim Norway

**Keywords:** educational attainment, genetics, policy, social science, sociogenomics

## Abstract

Research at the intersection of social science and genomics, ‘sociogenomics’, is transforming our understanding of the interplay between genomics, individual outcomes and society. It has interesting and maybe unexpected implications for education research and policy. Here we review the growing sociogenomics literature and discuss its implications for educational researchers and policymakers. We cover key concepts and methods in genomic research into educational outcomes, how genomic data can be used to investigate social or environmental effects, the methodological strengths and limitations of genomic data relative to other observational social data, the role of intergenerational transmission and potential policy implications. The increasing availability of genomic data in studies can produce a wealth of new evidence for education research. This may provide opportunities for disentangling the environmental and genomic factors that influence educational outcomes and identifying potential mechanisms for intervention.

**Key insights**

**What is the main issue that the paper addresses?**

This paper reviews the implications of recent discoveries in genomics for education research.

**What are the main insights that the paper provides?**

We describe how genetic data can be used to provide new insights into many research questions of interest, including understanding the causal mechanisms that link parents and offspring, and the influence of the environment on students’ outcomes.

## INTRODUCTION

A key aim of much social science research is identifying modifiable factors that can be used for interventions to improve people’s life outcomes. Our genomes are fixed at conception and cannot be changed, so why should education researchers – and social scientists more broadly – be interested in an aspect of human nature that is immutable? Here we discuss how researchers can exploit genomic data to enrich research in the social sciences.

Human behaviour is impacted by and associated with both genetic variation (differences in DNA between people) and environmental circumstances. However, genetics and the environment are not simply two independent forces; they are inextricably linked and jointly influence human behaviour (Plomin et al., 2014). Most human traits are heritable; that is, statistical variation in those traits is driven at least partially by genetic variation between individuals (Turkheimer, 2000, for a glossary see Box S1). If our aim is a holistic understanding of individual differences in educational or other social outcomes, it is important to incorporate genomics. At a practical level, inference from quantitative statistical approaches that fail to account for genomic information may lead to spurious conclusions about relationships between social factors that are in fact influenced by genomic differences between individuals. For example, parenting behaviour is related to many offspring outcomes, but parents and their offspring share half of their genomes and also often have similar social environments. Are these observed correlations driven by parental behaviours, by genetic correlation between parents and their children, or by a combination of both? Genomic evidence may not only capture the causal effect of genomics on education, but also reflect the effects of other social or familial processes.

The use of genomic data may also allow researchers to make stronger causal inferences than from other observational data. Suppose an investigator is interested in whether myopia (shortsightedness) affects educational attainment (discussed in Box 1), an example we will come back to later. Here, a correlation between myopia and attainment is not sufficient for concluding that myopia causes educational attainment, given the potential impact of confounding factors and reverse causation. Confounding could occur if a third factor affected both risk of myopia and educational attainment, while reverse causation could occur if processes leading to educational attainment (i.e. some aspect of the school environment) affect short‐sightedness. Under specific statistical assumptions, methods that exploit genetic variation can avoid these issues – which plague many observational studies – to reliably estimate causal effects. As with all empirical studies, however, a key consideration for interpreting genetic studies is whether the assumptions on which they depend are plausible.

Box 1 **The definition of educational attainment**

Many measures of the ‘years of education’ used in genome‐wide association studies (GWASs) like that of Lee et al. were derived from qualifications (e.g. in UK Biobank), making them a noisy and imperfect measure. This has implications, particularly for Mendelian randomisation (MR) (Howe et al., 2020). Furthermore, these measures of educational attainment differ across countries and were harmonised to a common scale (the International Standard Classification of Education [ISCED]). However, clearly there is going to be heterogeneity in what a given number of years of education mean in one country at one specific point in time vs. another country or even within the same country at different points in time. For example, consider two siblings, one has 17 years of education and the other 18 (starting at age 4 and leaving at age 21/22). One has a humanities degree from a traditional university, the other has a vocational degree from a newer university. Under the International Standard Classification of Education (ISCED) both siblings would be coded as having the same amount of education, but clearly the educational experiences and the downstream consequences of that education may differ. Similar arguments can be made about individuals leaving state vs. private education at the same age, or even choosing different subjects while at secondary school.

However, despite the heterogeneity in the definition of educational attainment, the signal from educational GWASs has been very consistently replicated within European ancestry samples. Therefore, the definitions of educational attainment are clearly sufficiently consistent across these samples to produce a reliable signal that explains around 10% of the variation. Fascinatingly, there is evidence that these signals do not replicate well in non‐European ancestry samples. For example, the association of educational attainment and the educational attainment polygenic score attenuates 85% in non‐European sample samples (Lee et al., 2018). This is probably because the score was developed largely in samples of European ancestry. However to date we do not have particularly good understanding or evidence about this because there are relatively few non‐European genetic samples compared with European samples (Mills & Rahal, 2019).

Researchers from education, economics, sociology and many other areas of social science have investigated the mechanisms which underlie the intergenerational transmission of education from one generation to the next. With the increasing availability of large datasets which have sampled mothers, fathers and offspring from the same families, researchers can use genetic variation to understand how educational advantage is transmitted within families.

While the genome is fixed at conception, the way that it affects educational outcomes may depend heavily on environmental circumstances. The opposite is also true: environmental impacts on individuals’ outcomes may depend on one’s genetic variation. Research in this area explores the interplay between genetic and environmental variation in shaping individual outcomes. Understanding whether the impact of different environments on educational outcomes differs across individuals with distinct genetic variation could help us to understand the existence of, causes of and development of educational inequalities within the population.

Below we provide an introduction to genetics for education researchers, discuss the relevance of recent findings in genomics to education researchers and how they can be used to understand the associations found in observational data, and finally explore the implications of these approaches for educational and social policies.

## GENOMICS 101

The human genome consists of DNA which is stored in our cells as chromosomes. Most humans have 23 pairs of chromosomes, one of each pair being inherited from each parent. DNA consists of two coiled chains of nucleotides that are bonded together in pairs and stored in a double helix shape. There are four types of nucleotides, defined by a difference in their base molecule: (A) adenine; (C) cytosine; (G) guanine; (T) thymine. There are around 3.2 billion of these nucleotide pairs in the human genome, but only a minority of these differ between people (The, 1000 Genomes Project Consortium, 2015). The points of nucleotide variation are referred to as single nucleotide polymorphisms (SNPs, pronounced ‘snips’), and different copies of these SNPs are referred to as alleles. Because our chromosomes come in pairs, for any specific SNP, we normally would have zero, one or two copies of an allele. Genomes can be measured from biological material such as blood or saliva, and their base pairs can be determined via genotyping.

## GENOME‐WIDE ASSOCIATION STUDIES

Genome‐wide association studies (GWASs) estimate associations between each SNP and a particular trait, or phenotype, such as educational attainment. Given the size of the human genome, a single GWAS can estimate millions of associations. Because so many associations are tested, GWASs use stringent ‘genome‐wide’ levels of statistical significance (*p* < 5 × 10^−8^) to test whether there is strong evidence that a SNP associates with a phenotype (Box 2). To further reduce the likelihood of false positives, many GWASs replicate their initial results in external, independent datasets, and then use a Bonferroni corrected *p*‐value for the number of SNPs exceeding genome‐wide significance levels in a replication sample (Box 2). An important consideration of GWASs is that they may be based on highly selected samples such as the UK Biobank, in which only 5% of people contacted chose to participate. If genetic variation is associated with participation in these studies, then GWAS results may not be representative of the underlying population (Taylor et al., 2018). Replication in more population‐representative external cohorts helps to reduce the risk of non‐representativeness, but it may still exist if replication cohorts are not fully population representative. GWASs can therefore be used to detect associations between specific traits and individual SNPs, but what relevance does this have for education and social scientific research?

Box 2 **Statistical significance in GWASs**

In the context of GWASs, the genome‐wide significance level has proven to be a useful indicator of association (Davey Smith, 2011). It is a relatively high bar, calculated on the basis of the number of independent variants in the genome, and the highest quality GWASs use a strict policy of replication in an independent sample. Thus, the threshold is 5 × 10^−8^ for discovery in the first sample, and then a Bonferroni corrected *p*‐value is used for the number of SNPs exceeding genome‐wide significance levels in a replication sample.

All that genome‐wide significant signals from GWASs tell us is that there is evidence that a genetic variant is associated with a phenotype in the discovery and replication samples. This does not demonstrate that a genetic variant causes the phenotype in an individual, or is necessarily associated in the population of interest. There are a number of processes that could induce associations between SNPs and phenotypes, only one of which is a direct causal effect of the variant on the phenotype. Samples which are selected on the phenotype of interest and other traits (e.g. educational attainment and body mass index, BMI) may induce spurious correlations between SNPs causally associated with BMI and educational attainment and vice versa (i.e. selection/collider bias). Equally, population structure or familial effects can induce these associations. Therefore discovery that a genetic variant associates with a phenotype like educational attainment is really the first step in a scientific process; it is nevertheless an absolutely necessary (but not sufficient) step for elucidating the causal links between the genetic variant and a phenotype like educational attainment.

The latest GWAS of educational attainment, defined as years of completed education (Lee et al., 2018), included 1.1 million individuals. It identified 1271 education attainment‐associated SNPs at genome‐wide significance. These results have been replicated across multiple samples and demonstrate that common genetic variation associates with educational attainment. These associations may reflect biological effects of genetic variation on biological, social and psychological phenotypes that ultimately affect educational attainment. However, a substantial fraction of these associations are likely to reflect demographic and familial processes such as assortative mating (i.e. that spouses tend to be similar; see Box S1 for a glossary) and effects of parents on their children (i.e. parenting; Brumpton et al., 2020; Davies, Howe, et al., 2019). Thus, genetic data can provide compelling evidence not only about purely biological effects, but potentially about the impact of parents, the familial environment and society.

An important finding from GWASs of education and other social/behavioural phenotypes is that they are highly ‘polygenic’. That is, these phenotypes are characterised not by a small number of SNPs with large effect sizes, but by a very large number of SNPs which each have tiny, but statistically detectable, effect sizes. Critically, *there is no such thing as a ‘gene for’ education*; of the 1271 education attainment‐associated SNPs, the median SNP association was only 1.7 weeks of additional education throughout the lifecourse (1.1 and 2.6 weeks for the 5th and 95th centile SNPs; see Box 1 for a discussion on measuring education). This pervasive collection of tiny effects – which has been discussed for over a century – has recently been labelled the omnigenic model (Boyle et al., 2017).

Such small effect sizes can only be detected in very large samples. More powerful predictive models can be constructed by aggregating individual SNPs into a polygenic score, which is a weighted average of the associations of the genetic variants and the trait. While individual SNP effects can be small, when combined into a polygenic score they can explain substantial amounts of trait variation. For example, the best performing polygenic score of the most recent education GWAS explains 11–13% of variation in educational attainment (Lee et al., 2018).

## SNP HERITABILITY AND GENETIC CORRELATION

Researchers can use GWAS results to estimate the proportion of variation in a trait such as educational attainment that can be explained by measured genetic variation.^1^ This estimate is termed the ‘SNP heritability’. Studies may report the proportion of variation in a trait explained by SNPs that were genome‐wide significant, or all measured SNPs that were associated with the phenotype of interest, irrespective of statistical significance. The former approach tells us how much of the variation in a trait can be explained by variants with established effects, while the latter indicates how much variation can be explained by all measured genetic variation, even where it is noisily measured. Because there are fewer genome‐wide significant SNPs, they typically explain far less of the variation in a trait than estimates using all SNPs; 2–3% of the variation in educational attainment vs. 11–13% in the most recent education GWAS (Lee et al., 2018). Heritability estimates from genomic studies are typically smaller than those from twin studies because they rely on measured rather than total genetic variation.

Genetic correlations, the correlations between genetic associations with one trait and a second trait, can also be estimated from genomic data. There are two commonly used methods for estimating genetic correlation, although they can also both estimate heritability as well. The first of these, called LD‐score regression (Bulik‐Sullivan et al., 2015), uses large‐scale GWAS data. The second, called Genome‐wide Complex Trait Analysis using Genome‐based Restricted Maximum Likelihood (GCTA‐GREML; Yang et al., 2011), uses genetic relatedness between individuals which is derived from individual‐level genetic data. The intuition for GCTA‐GREML is that if genetic variation explains (some of) phenotypic variation, individuals who are more genetically similar are expected to be more phenotypically similar (analogous to twin studies).

**FIGURE 1:**
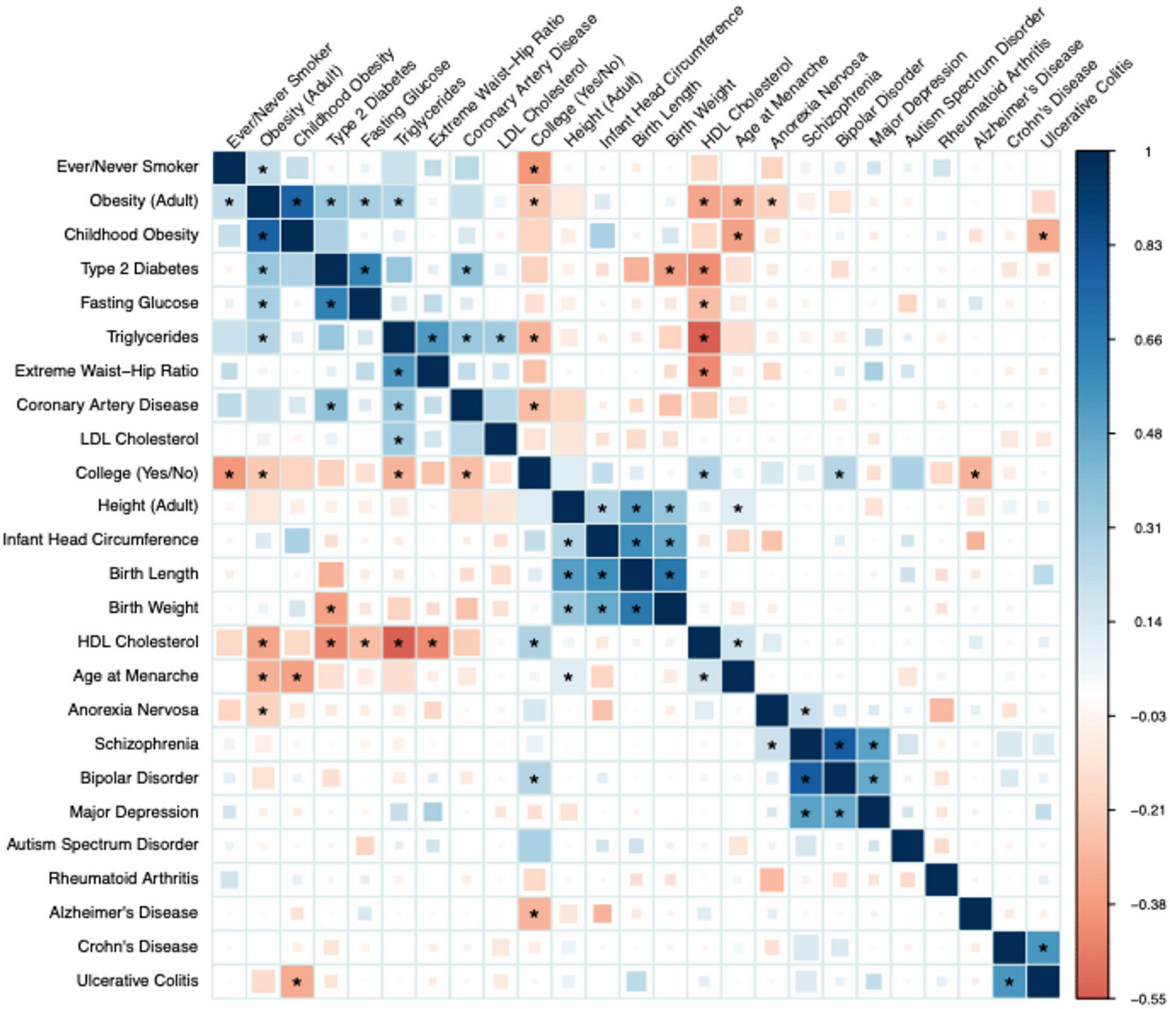
Genetic correlations from 25 genome‐wide association studies. Squares represent the genetic correlation between two traits; blue represents positive genetic correlations while red represents negative genetic correlations. Coloured square size corresponds to *p*‐values for tests of difference from zero (no genetic correlation), with larger squares denoting smaller *p*‐values. Full‐size squares denote values that pass a false discovery rate of 0.01, while squares with an asterisk denote values that pass Bonferroni correction for the 300 tests conducted. Educational attainment was measured in this study using the variable ‘College (yes/no)’. This indicates that genetic variation associated with graduating from college is also associated with being less likely to be a smoker, to be obese, to have lower triglycerides and LDL cholesterol, to have higher high‐density lipoprotein cholesterol and to have lower liability to bipolar disorder and Alzheimer’s disease. Reproduced with permission from Bulik‐Sullivan et al. (2015). [Colour figure can be viewed at wileyonlinelibrary.com]

Studies have shown that higher educational attainment‐associated genetic variation is also correlated with lower levels of smoking, lower body mass index (BMI), lower risk of coronary heart disease, lower triglycerides, higher high‐density lipoprotein, being taller and being at higher risk of bipolar disorder (Figure 1). These results show that the genetic correlations between genetic variation related to one trait, such as BMI, and another trait, such as educational attainment, tend to be similar to their associations in other forms of observational studies. They also suggest that many genetic variants are likely to affect multiple phenotypes (referred to as pleiotropy).

The correlogram in Figure 1 illustrates the genetic correlations between a range of traits. This includes the genetic correlations between variants associated with attending college, from one of the first GWASs of educational attainment by Rietveld et al., and a range of other traits (Rietveld et al., 2013). Each of the squares in the figure represents a genetic correlation between two traits, e.g. attending college and being an ever/never smoker. This figure illustrates that genetic variants that associate with attending college also associate with a range of health traits. This therefore provides evidence of widespread pleiotropy (i.e. genetic variants that associate with multiple traits; Verbanck et al., 2017).

## GENES AND ENVIRONMENTS

Researchers can use genetic data to explore the presence of gene–environment correlation (*rGE*). *rGE* refers to the fact that certain environments are more prevalent for carriers of certain genetic variants and can arise from three mechanisms (Plomin et al., 1977). First, *evocative* gene–environment correlation occurs when an individual’s genetic variation evokes specific responses from others, creating a correlation between the individual’s genetic variants and their environment. For example, having a high polygenic score for educational attainment (which may manifest in certain learning attitudes and behaviours) may invoke different responses from parents and teachers, whereby they create more learning opportunities.

Another form is *active* gene–environment correlation, which refers to the way that an individual can select their environment based upon their genetics. For example, are children with a genetic predisposition for creative activities more likely to select certain subjects or activities? This is an active choice from the individual, which is correlated with their genetics.

Finally, there are *passive* gene–environment correlations, which often arise owing to the inheritance of both genetic variation and environments from one generation to the next. For example, the offspring of parents with high polygenic scores for education are also themselves likely to have high polygenic scores for education owing to direct genetic inheritance. If these parents create educational nurturing environments because of their genetics, the offspring are likely to also inherit environments that improve their learning and education. Hence, the genetic variation and environment that offspring inherit are correlated, a phenomenon known as ‘genetic nurture’ (see e.g. Bates et al., 2018; Kong et al., 2018; Plomin & Bergeman, 1991; Wang et al., 2021; Wertz et al., 2018).

One way social science research has used genomic data is via studies of gene‐by‐environment interplay which aim to quantify the way that genetic effects may vary with environmental context and vice versa (Box 3). Existing studies of gene–environment interactions have for example shown that the social environment moderates genetic vulnerability to poor socioeconomic outcomes (see e.g. Belsky et al., 2016, 2018; Meyers et al., 2013; Papageorge & Thom, 2020; Rutter, 2006; Shanahan et al., 2008). A challenge in these studies is having sufficient statistical power (i.e. large enough sample sizes) to reliably detect interactions. Many of the most celebrated (and cited) ‘interactions’ claimed in the behavioural and psychiatric genetic fields have failed to replicate (Duncan & Keller, 2011).

Box 3 **Gene–environment interactions and correlations**

The latest GWAS by Lee et al. (2018) shows that genetic variation explains a substantial amount of variation of educational attainment. Environmental or family circumstances, such as parental time and monetary investments in children, are also important. A study of the gene–environment interplay can shed light on how these two interact in shaping educational outcomes. Indeed, Muslimova et al. (2020), Howe et al. (2021) and Demange et al. (2020) build on a well‐known literature that shows that firstborns, on average, have more years of education than later‐born siblings, probably owing to additional parental investments in the early years. Muslimova et al. (2020) used a sample of siblings from the UK Biobank, where both birth order and genetic variation are random. They show that siblings with a higher polygenic score benefit more from being firstborn than those with a lower polygenic score. Both one’s birth order and one’s genetic variation are random within families, meaning that the analysis explores the causal effect of such gene–environment interactions.

However, it is often unclear what may be driving the interplay between genes and environments: does one’s genotype truly moderate the environmental effect, or is the interaction effect caused by gene–environment correlation? Suppose we are interested in the effect of the polygenic score for education on years of schooling. In the presence of genetic nurture, the polygenic score will partially capture environmental and familial effects shaped by the parental genotype (i.e. passive *rGE*). If the direct genetic effect and the familial effect operate in the same direction, this will cause upwards bias in the genetic effect, leading us to believe that the direct effect is larger than it truly is. Including an additional interaction effect of the polygenic score and the environmental factor may also overestimate the coefficient of interest owing to this *rGE*.

The use of family genetic data can address this problem directly as genetic variation is random between siblings and for offspring, conditional on parental genetic variation. This means that one can identify the causal direct genetic effect by exploiting within‐family genetic designs that examine between‐sibling differences or control for parental genotypes. Again, however, a challenge in these studies is statistical power.

## CONFOUNDING, BIAS AND CAUSATION

Disentangling cause and effect from correlation is notoriously difficult. Bias owing to confounding factors or reverse causation impedes many studies, making it difficult to reliably draw causal inferences from observational data. Suppose a researcher is interested in the relationship between myopia (shortsightedness) and educational attainment. Correlations between these variables may arise because of a causal effect of myopia on education, a background factor that causes both of these variables (such as time spent reading) or a reverse causal effect of education on myopia. Ordinary least squares regression techniques commonly applied to observational data are unable to distinguish directionality (causation vs. reverse causation), while causal effects can only be estimated if the regression model includes a sufficient set of confounder variables which are measured without error.

**FIGURE 2:**
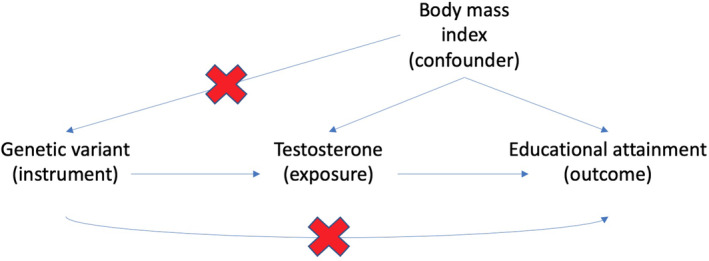
A directed acyclic graph illustrating the relationships between the independent variable (shortsightedness), dependent variable (educational attainment) and instrument (the genetic variant). The association of shortsightedness and educational attainment is potentially biased by socioeconomic position. Genetic variation in shortsightedness is unlikely to be related to socioeconomic position, is fixed at conception and cannot be affected by either shortsightedness or educational attainment. We can use these genetic variants to estimate the effect of shortsightedness on educational attainment (Mountjoy et al., 2018). One of the key strengths of MR is in demonstrating that some mechanisms *are unlikely* to be causal. [Colour figure can be viewed at wileyonlinelibrary.com]

Under specific assumptions, researchers can exploit genomic data to provide more reliable causal evidence compared with methods that attempt to measure and adjust for confounders (e.g. multivariable adjustment or propensity score approaches). Mendelian randomisation uses the random inheritance of genetic variation from parents to offspring as a natural experiment to strengthen causal inference with respect to potentially modifiable influences on health and development (Davey Smith & Ebrahim, 2003; Davies et al., 2018; Richmond & Davey Smith, 2021, Sanderson et al., 2022). Mendelian randomisation can be implemented as an instrumental variable (IV) analysis, with the SNP (or SNPs) acting as an IV for the exposure under investigation. The IV methods depend on three instrumental variable assumptions: (a) that the instrument associates with the independent variable of interest (relevance); (b) that there are no factors that affect both the independent (exposure) and dependent (outcome) variables (no confounding/independence); and (c) that there are no direct paths from the instrument to the dependent variable except via the independent variable of interest (the exclusion restriction). Figure 2 illustrates these assumptions graphically.

Regarding the relationship between myopia and educational attainment, observational studies have found evidence that, on average, more short‐sighted people remain in school for longer. However, it is not clear whether this is because learning‐associated factors (such as reading books) affect people’s eyesight or whether shortsightedness affects people’s progress throughout education systems (Ip et al., 2008). The policy implications of these two hypotheses are very different. If short‐sightedness affects educational attainment, then steps to address students’ eyesight could lead to a fairer education system. If something about the educational process affects the risk of myopia, then there might be ways to mitigate this risk (e.g. spending more time outdoors; Guggenheim et al., 2012). While observational studies will not provide evidence about the causal effect driving this association, genomic studies can provide more compelling evidence about the direction of the effect. For example, we could investigate the associations of myopia‐associated SNPs with educational attainment and the associations of education‐associated SNPs with myopia to disentangle the direction of causation. If myopia affects educational attainment, we would expect individuals who inherit SNPs associated with myopia to have different educational attainment from individuals who do not inherit them. Conversely, if something about the educational process affects the risk of myopia, then we would expect that individuals who inherit education‐associated SNPs to be more likely to develop myopia than individuals who do not inherit them (Figure 2). Mountjoy et al. (2018) investigated this hypothesis and found compelling evidence that myopia‐associated SNPs did not strongly associate with educational attainment but that education‐associated SNPs associated with myopia. These results suggest that the direction of causation runs from some process leading to educational attainment to myopia, rather than vice versa. Studies have been conducted for a wide range of other hypotheses and risk factors and can in theory be applied to a wide range of educational and social scientific traits where GWASs have been conducted.

### Assessing the instrumental variable assumptions

The key to interpreting evidence from genetic studies is to determine whether assumptions on which they depend are plausible (Doidge & Dearden, 2017; Hemani et al., 2018a). GWASs have identified genetic variants that consistently associate with many traits of interest, so the first instrumental variable assumption (relevance) is likely to hold and can be assessed.

At conception, at each point of variation in the genome, offspring randomly inherit either their mother’s or their father’s DNA. DNA is set at conception, and so generally the environment does not affect germline genetic variation during the life course – a 90‐year‐old has the same DNA they were born with. This makes reverse causation unlikely (e.g. an outcome such as myopia, affecting genetic variation associated with educational attainment).

Furthermore, the random allocation of genetic variation at conception means that many, but not all, genetic variants are randomly distributed across the population. Hence the second instrumental variable assumption (independence) may hold, although this should always be evaluated.

It is not possible to statistically prove that the genetic variants only affect the outcome via the proposed exposure, i.e. that the exclusion restriction holds. However, under the assumption that exposure has a constant effect on the outcome, it is possible to falsify this assumption by testing whether different genetic variants imply different effect sizes. If there is substantial heterogeneity in the estimates, then this suggests that a simple linear effect of the exposure on the outcome is unlikely, or that (one of) the instrumental variable assumptions do(es) not hold.

**FIGURE 3:**
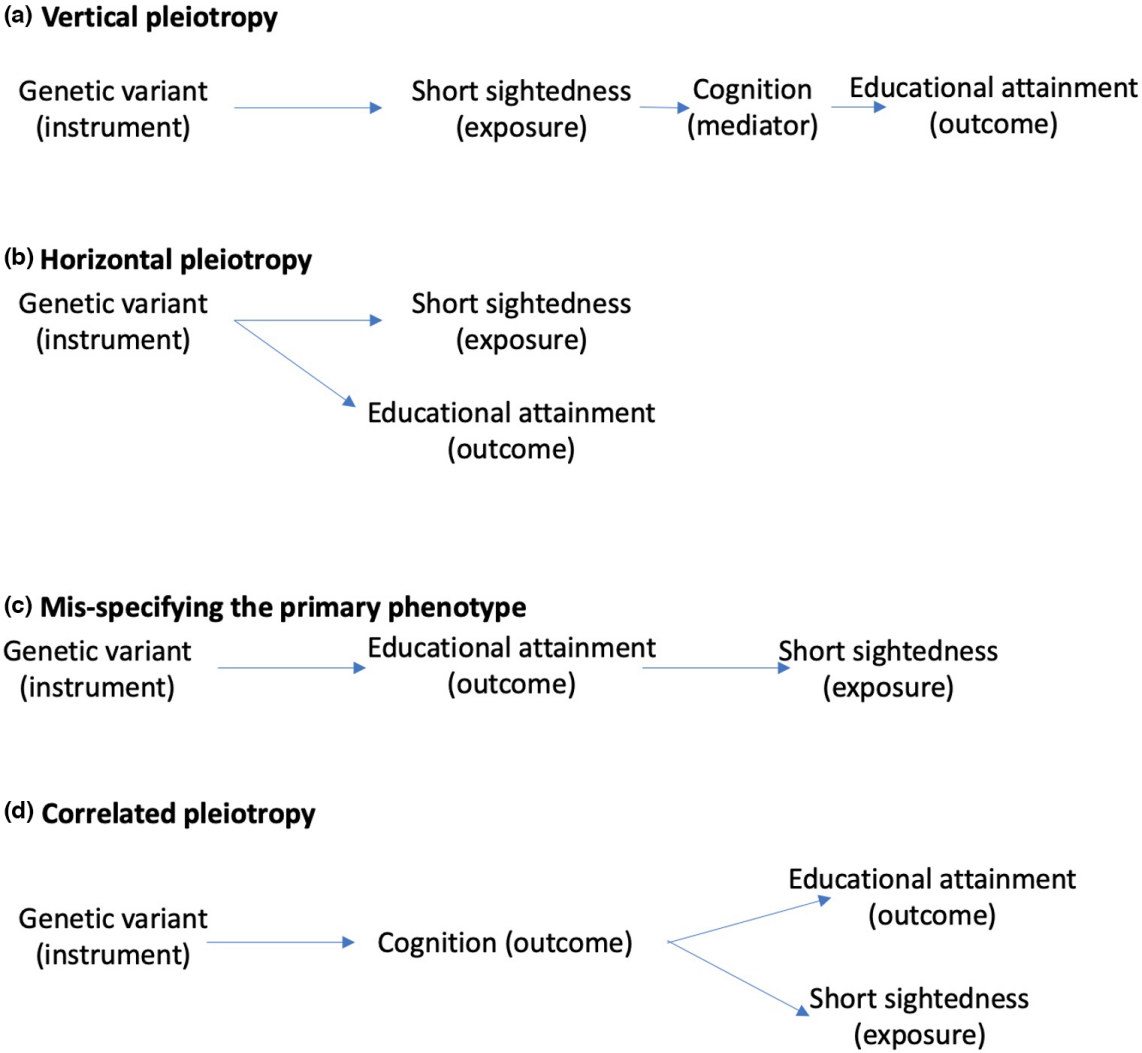
Possible structure of pleiotropic effects. In (a) the genetic variant affects short‐sightedness, which in turn affects cognition, which in turn affects educational attainment. Vertical pleiotropy does not violate the assumptions of Mendelian randomisation (MR) here because educational attainment is purely a downstream factor from cognition. In contrast, in (b) if the genetic variant affects the exposure and outcome through distinct pathways then MR will be biased because horizontal pleiotropy violates the third instrumental variable assumption (exclusion restriction). Here, the variant can independently affect the outcome through the exposure or a third variable. In (c) a MR analysis which mistakenly identifies the primary phenotype influenced by the genetic variant used as an instrument is shown. If the instruments for education are considered to be instruments for a downstream trait influenced by education – such as short‐sightedness – then inference will be in the wrong direction. This can be assessed using Stieger filtering (Hemani et al., 2018a). In this situation the pleiotropic effects will correlate with the effects through the exposure of interest, and such correlated pleiotropy will distort findings even when using many of the sensitivity analyses that have been developed for MR (Richmond & Davey Smith, 2021). [Colour figure can be viewed at wileyonlinelibrary.com]

The exclusion restriction requires that instrumental variables only affect the outcome via the exposure of interest, i.e. that myopia‐associated genetic variants only affect the outcome (educational attainment) via their effects on myopia. This may be plausible for genetic variants related to myopia, but it is more challenging for studies investigating the effects of educational attainment as an exposure. Educational attainment is generally considered to be a phenotype more distal from the genome than is myopia; therefore genetic variants that relate to educational attainment are likely to have more pleiotropic effects (i.e. the genetic variants are likely to be associated with other traits, such as cognition). Pleiotropy can be vertical, where a genetic variant affects one trait (e.g. cognition), which then affects an outcome (e.g. educational attainment). As long as the primary phenotype which the genetic variant relates to is correctly specified, then vertical pleiotropy is the essence of MR (Figure 3a; Davey Smith and Hemani 2014). Alternatively, pleiotropy can be horizontal, when a genetic variant affects both an exposure under investigation (e.g. educational attainment) and other variables through independent pathways. If one of these independent pathways influences the outcome (e.g. short‐sightedness), then this violates the exclusion restriction and causes bias in MR studies (Figure 3b).

If variants that primarily influence an upstream phenotype are mistakenly taken to be instruments for the downstream phenotype then inference will be incorrect (Davey Smith and Hemani 2014). For example, if a presumed genetic instrument for education is in fact primarily an instrument for cognition, then MR will estimate the causal effect of cognition – which includes any downstream effects acting through education – on the outcome. Methods exist that can improve the correct identification of the most proximal phenotype to a genetic variant (Hemani et al., 2017) (Figure 3c) Particularly problematic is when a genetic variant influences a confounder of the exposure to outcome association (Figure 3d). In this situation the pleiotropic effects will correlate with the effects through the exposure of interest, and such correlated pleiotropy will distort findings even when using many of the sensitivity analyses that have been developed for MR (Richmond & Davey Smith, 2021).

There are multiple methods which seek to evaluate the structure of pleiotropy and estimate the effects of exposure, even if some or all of the genetic variants have horizontally pleiotropic effects, or to evaluate the effects of multiple pathways, for example educational attainment and cognition, such as Anderson et al. (2020), Davies, Hill et al. (2019) and Sanderson et al. (2019). These use standard multivariable instrumental variable estimators to estimate the effects of multiple exposures using multiple instruments, e.g. see Greene (1993) and Wooldridge (2002).

Horizontal pleiotropy is probably less of an issue for more biologically proximal phenotypes, and may therefore have less of an impact on studies of some putative exposures that affect education (such as myopia) than studies of educational attainment as an exposure itself. This is doubly true if an exposure is found to have a precise null effect on educational attainment, such as in the example of shortsightedness. Horizontal and correlated pleiotropy are plausible explanations for why we might see associations between genetic variants known to associate with a trait like educational attainment and other outcomes such as myopia (i.e. false positive findings).

Mendelian randomisation requires a further fourth ‘point identifying’ instrumental variable assumption. This assumption can include that the exposure has a constant effect on the outcome or that the instrument (genetic variant) has a monotonic effect on the exposure; for more on this see Howe et al. (2021, 2021b).

### Weighing evidence from genetic and randomised studies

The Educational Endowment Foundation (an education research funder in the UK) has funded a randomised trial of 700 students across 100 schools to estimate the impact of eyeglasses on educational outcomes for 4‐ and 5‐year‐olds who need them (Educational Endowment Foundation, 2021). This project will provide gold standard evidence for whether encouraging children to wear glasses can improve their reading achievement, mathematics achievement and visual acuity. The genetic evidence presented in Mountjoy et al. (2018) suggests that interventions to increase the use of glasses to overcome myopia (i.e. shortsightedness) are unlikely to substantially increase lifetime educational attainment (that is, the years of school attained).

However, there are crucial differences between the outcomes and exposures in the randomised trial and the genetic study. The exposure in the genetic study is myopia, whereas the intervention in the randomised trial covers a number of visual conditions, including short and long sightedness, amblyopia (lazy eye) and/or strabismus (a squint). The outcome in the genetic study relates to educational attainment, whereas the primary outcome for the trial is reading achievement measured at the end of the first year of school. Thus it is perfectly possible that the trial finds evidence of short‐term increases in educational achievement in spite of the genetic evidence. Ultimately, the closest comparison will be whether this trial affects educational choices in 14 years when participants are aged 18+. This quite neatly highlights one of the strengths of genetically informed designs – that we can potentially provide some evidence about very long‐term effects of interventions like glasses, that would take an enormously long time to discover using randomised controlled trials. However, it also highlights one of the limitations, that the results of genetic analysis estimate the effects of a specific exposure (myopia in the case of Mountjoy et al.) and a specific outcome (educational achievement/years of education). Both may differ from the hypothesis of interest, e.g. does encouraging 4‐ to 5‐year‐olds to wear glasses affect how well they learn to read?

### Controlling for genetic variation

Genetic variables generally make very poor covariates. This is because they typically only explain a small proportion of the variation in a trait, and we know that there is almost certainly genetic variation that we have not identified (this is implied by the omnigenic model and limited statistical power of GWASs to identify all relevant genetic variation). Therefore, studies rarely control for genetic variables, and it is rarely a plausible or useful study design. There have been sophisticated attempts to combine instrumental variable analysis and genetic variation to, in effect, control for the mismeasured or unmeasured genetic variation contributing to a trait (most notably DiPrete et al., 2018; van Kippersluis et al., 2021) and repositories of polygenic indices (preprint: Becker et al., 2021; Richardson et al., 2019).

## INTERGENERATIONAL TRANSMISSION

Given the predominance of data collection focussed on individuals rather than families, genomic data has historically only been available for unrelated individuals in large numbers. However, cohort and longitudinal studies are increasingly collecting genomic data on families, which offers new opportunities for studying the molecular and social mechanisms that mediate the intergenerational transmission of phenotypes such as educational attainment. For example, GWASs have suggested that the effects of SNPs that associate with educational attainment may be mediated via the effects of parents on their offspring. It is also possible to investigate the environmental mediation of these education‐associated SNPs through familial and social factors, such as parenting behaviour. Recent research has demonstrated that SNP‐educational attainment associations are at least partially mediated by environmental factors (Howe et al., 2021; Kong et al., 2018; Selzam et al., 2019). That is, estimated genetic associations do not represent just biological effects, but also social and demographic processes such as assortative mating and indirect effects such as genetic nurture. For example, if people who are more educated tend to have children with people who have lower BMI, then genetic variation that affects BMI and educational attainment will be correlated in their offspring. Similarly, if parents’ BMI affects their offspring’s educational attainment, then we would expect to see associations between genetic variation that affects BMI and the offspring’s educational outcomes, even if BMI does not affect educational attainment at the individual level.

**FIGURE 4:**
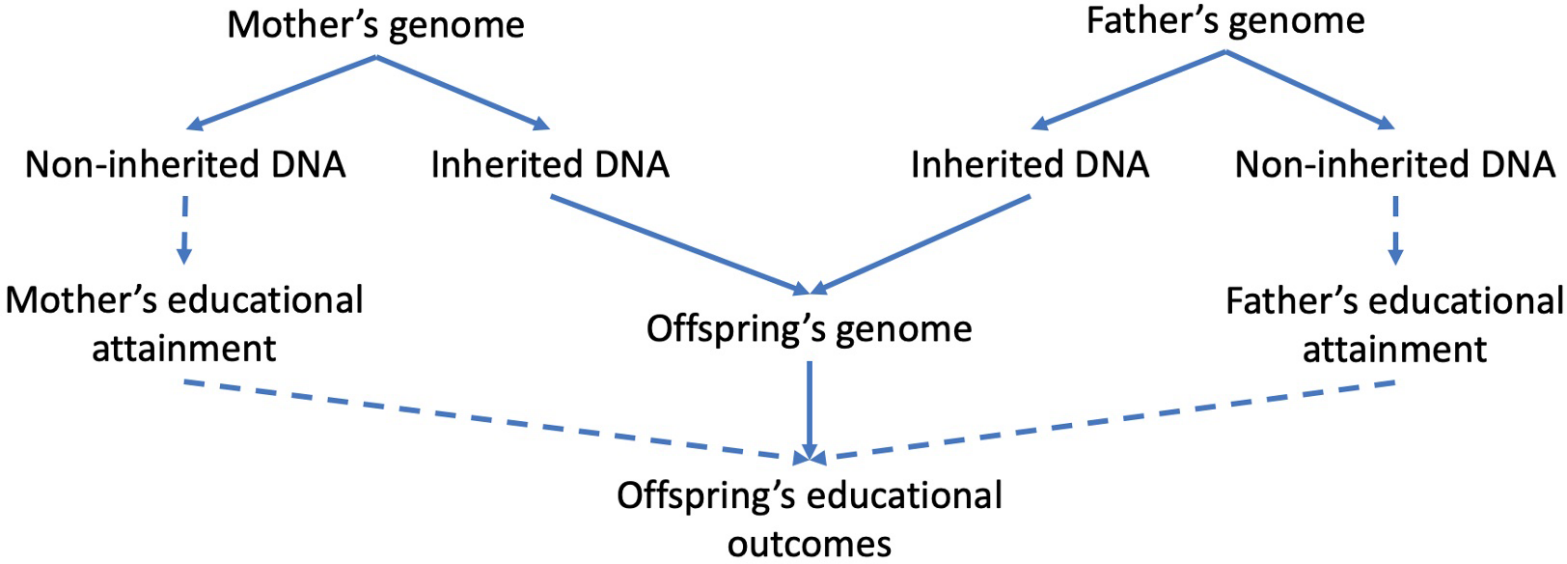
Relationship between parents’ educational attainment and offspring educational attainment. The non‐inherited genetic variation can be used to estimate the effects of parental education (or other parental traits) on offspring educational outcomes free from confounding via direct genetic inheritance. [Colour figure can be viewed at wileyonlinelibrary.com]

The half of parents’ genomes that a child doesn’t inherit, the ‘non‐inherited DNA’, can also be used to investigate environmental mediation of genetic effects. Here, one can estimate parents’ effects on their children through pathways other than direct genetic inheritance (Figure 4). Because these SNPs are not inherited, they cannot have a molecular effect within offspring and must therefore only impact offspring through the environment. These effects could include the impact of the family environment, such as parenting styles or parental personality, or population‐level phenomena such as assortative mating. Hence, perhaps counter‐intuitively, incorporating *genetic variation* in educational analysis can allow researchers to directly explore the role of the *family environment* in shaping offspring outcomes.

## EDUCATION POLICY IMPLICATIONS

There has been a debate about the potential use of genetic data for educational policy (Grigorenko, 2007; Sabatello, 2018). The data and software resources are increasingly available (see Boxes 4 and 5). Some have argued that the presence of genetic associations with education and education‐associated phenotypes indicates that educational policies should be tailored to individuals (Plomin, 2019; von Stumm & Plomin, 2021). This could be by identifying individuals with low genetic liabilities for education before entering the education system and targeting specialist education interventions to support their learning and address learning inequities.

Box 4 **UK genetic data available for education researchers**

- Avon Longitudinal Study of Parents and Children – a longitudinal birth cohort study that sampled births between 1990 and 1992 in the Avon area. Large sample of mother and offspring genetic data, with a small number of samples from fathers. Questionnaire data and linkage available to the National Pupil Database, a census of exam results taken in UK state schools.
- Generation Scotland – large family based sample including genetic and epigenomic data. Extensive questionnaire, cognitive ability and linkage data.
- Millenium Cohort study – nationally representative birth cohort which also sampled some family members (i.e. parent–offspring trios). Data available on educational attainment and linkage to the National Pupil Database.
- National Child Development Study (1958) – representative cohort of individuals born in a single week in 1958 in the UK. Educational data collected during childhood and very long follow‐up of health outcomes. Genetic data generated in a number of different samples, quality‐controlled processed data will be available soon from the Centre for Longitudinal Studies.
- UK Biobank – a very large cohort study of nearly 500,000 individuals. The study has limited questionnaire data on educational attainment, how long participants remained in school and their qualifications. It has detailed health, biomarker, imaging data and linkages to medical records.
- Understanding Society (UKHLS) – household panel survey with genetic data on up to 12,000 individuals sampled by household.

Box 5 **Resources for researchers**

**METHODS**

There are a wide range of textbooks available that provide a detailed introduction to the use and analysis of genomic data (Asbury & Plomin, 2014; Mills et al., 2020) and reviews of the use of genetic data and educational outcomes (Freese, 2018; Plomin & von Stumm, 2018). The European Commission, Joint Research Centre (2019) report into social science genomics provides an excellent overview of recent developments in genomics and their implications for policy‐makers. There are also a number of published reviews on MR and other genetic approaches, for example Davies et al. (2018) and Richmond & Davey Smith (2021) and specifically applied to familial data (Davies, Howe et al., 2019; Hwang et al., 2020; Thapar & Rice, 2021). An edited volume on a wide range of causal inference approaches involving human genetics is Davey Smith et al. (2021). Evans et al. (2021) published a special issue of *Behaviour Genetics* on describing methods for the genetic analysis of complex traits. Cesarini and Visscher (2017) provide an excellent summary of the potential uses of genetic evidence for educational research, building on earlier papers (Beauchamp et al., 2011; Benjamin et al., 2012). The Social Science Genetic Association Consortium (SSGAC) Summer Institute in Social Science Genomics provides an extensive reading list and lectures (SSGAC, 2021).

**SOFTWARE**

Genetic data can be used in educational research without specialist software. For example, if you have data on a specific genetic variation, i.e. how many alleles (zero, one or two) each participant has at a particular locus, these variables can be included in regression or other models in the same way as any other variable. However, many genetic datasets are provided in bespoke formats for genetic data. This is because there are a lot of variables (up to 80 million), and most standard formats (e.g. csv, Stata, SPSS, or R data files) struggle to handle this many variables. Common genetic data formats include.vcf (variant call format) and plink format (.bim,.bed and.fam for the summary information about the genetic variants, the genetic data for each participant and the ID information respectively). These files need to be processed with specialist software such as Plink (Purcell et al., 2007). Genetic correlations can be estimated using software such as GCTA (Yang et al., 2011). There are also specialist software packages and databases for running MR analysis such as the TwoSampleMR package for R and OpenGWAS (Hemani et al., 2018b).

However, there are two major issues with genetically informed policies aimed at individuals, such as using genomic information as a basis for school entry selection. First, there is growing evidence that observed SNP associations with education also reflect family and demographic factors as outlined above (Howe et al., 2021a; Kong et al., 2018; Morris, Davies, Hemani et al., 2020; Wang et al., 2021). This evidence suggests that polygenic scores are likely to reflect not only the causal effects of SNPs on education, but also the influence of other social and familial processes. This may not matter if the goal is the prediction of general educational performance, but may matter if the goal is to identify specific causal pathways for support or to understand mechanisms, because non‐causal relationships will not make good intervention targets. Second, genetic data currently add very little knowledge to individual‐level predictions above other data that educators already have access to, such as age in year, sex and indicators of socioeconomic position such as free school meals (Morris, Davies & Davey Smith, 2020). Polygenic scores are likely to be useful for understanding relationships at the population level, but it is less clear that they will be useful for providing individual‐level predictions and personalised education (Davey Smith, 2011). Furthermore, genetically informing educational policy at the individual study level is unlikely to be politically acceptable or feasible. For further details see European Commission, Joint Research Centre (2019).

Genetically informed policies aimed at the group level, such as interventions designed to support pupils’ progress, are probably more feasible. Genomic data also offers policy promise through how it can be used to assess structural differences within educational systems under differing confounding structures to traditional social scientific data. For example, genetic data can be used to investigate the benefits of studying at more advantaged schools net of genetic liability (Harden et al., 2020), selection differences between schools by socioeconomic position (Trejo et al., 2018), the differential performance of students with similar genetic liability for education from advantaged and disadvantaged backgrounds (Stumm et al., 2020), the potential role of genetics in fostering intergenerational social mobility (Belsky et al., 2018) and the robustness of value‐added progress measures to pupil‐level factors (Morris et al., 2018).

## ETHICS

There are a number of ethical risks and concerns to the use of genetic data in educational research and policy. Schools currently use a wide array of data to select students for entry, group formation, targeted interventions or even exclusion. Children may respond to knowledge about their genomes (Van Wietmarschen et al., 2006) in ways that affect their learning, for example, by reducing their self‐esteem (Kuther, 1994). There are also ethical issues surrounding informed consent from children and governing access to their genetic data. Velarde et al. (2021) argue that citizens should be able to decide ‘who and how to trust’ with their genetic information.

Many existing genetic studies have a Eurocentric approach (Mills & Rahal, 2019) and polygenic scores do not perform well across populations of different ancestries (Duncan et al., 2019). This means that inequalities between groups could be further entrenched by the systematically unequal benefit of genetic applications to European‐ancestry populations. Furthermore, isolating genetic effects from social or environmental effects may be difficult given the social stratification that exists in many societies and the differential experience of environments between different groups (Martschenko et al., 2019). This is contextualised by an ugly history of eugenics that underlies some genetic research into intelligence in the nineteenth and twentieth centuries (Martschenko et al., 2019). Elements of this continue in the form of race pseudoscience that confuses or obfuscates genetic ancestry with race or ethnicity. Ancestry is a process‐based concept that refers to the genetic similarity of individuals to one another that can be identified using genetic data (Yudell et al., 2016). Race is a pattern‐based concept rooted in oppression that refers to the way that individuals have historically been ascribed to socially constructed or geographically defined groups and cannot be identified using genetic data. Ethnicity is a broad pattern‐based approach rooted in identity that commonly refers to a broad range of shared factors such as traditions, cultural experiences or national origins, and cannot be identified using genetic data. For further information, see Birney et al. (2021), who provide a detailed discussion and guidance about the importance of the scientific language used to describe research using genetics.

## SUMMARY

Findings from sociogenomics have the potential to improve our understanding of not only the genomic and biological factors, but also familial and social factors that combine across the life course to affect educational outcomes. Incorporation and uptake of genomic data may help to inform policymaking and the better design of interventions. This may be through identifying how nature and nurture interact to shape individual outcomes, elucidating the causal mechanisms underlying educational outcomes, and by providing novel insights into the transmission of educational advantage across generations. Many insights from genetics for education research are likely to be made at the population level (i.e. factors or processes that affect people on average), rather than for individual‐level prediction (i.e. personalised education). With that, genetic data may be able to provide a wealth of evidence for education research.

## ETHICS STATEMENT

This review did not conduct any new empirical research and did not require ethical approval.

## CONFLICT OF INTEREST

We receive, or have received funding for research into the use and application of genetics.

## Supporting information

Supplementary Material

## Data Availability

Data sharing not applicable to this article as no datasets were generated or analysed during the current study.

## ENDNOTE

^1^
For example, this is effectively the *R* ^2^ of genetic variation for the trait.
