# Supplementary material for: Implications of the genomic revolution for education research and policy
Source: Br Educ Res J. 2022 Apr 12;50(3):923–43. doi: 10.1002/berj.3784 (PMC11225938; doi:10.1002/berj.3784)
Supplement: Supplementary file 1 — Supplementary Material [file BERJ-50-923-s001.docx]

**Supplementary Box 1: Glossary (Morris et al. 2018)**

**Allele**

Alleles are the different variant forms of genetic variation found at a specific point on a chromosome. Specific alleles associate with different phenotypic traits (e.g. outcomes such as educational attainment).

**Allele frequency**

Allele frequency is the prevalence of a given allele at a genetic locus in the sample, expressed as a decimal, fraction or percentage. The allele frequency is reported in terms of the proportion of alleles that are the effect allele (e.g. the allele associated with higher levels of education) or in terms of the number of minor, or less frequent, alleles (termed minor allele frequency).

**Assortative mating**

Assortative mating refers to the non-random manner in which people sort into partnerships with partners who have more similar social and biological characteristics such as height, education and personality than would be expected by chance alone. This may be due to assortment based upon partner choice, convergence in characteristics due to interaction with a partner over time or social homogamy (Robinson et al., [2017](https://bera-journals.onlinelibrary.wiley.com/doi/full/10.1002/berj.3466#berj3466-bib-0030)). Rates of assortative mating may vary between populations and over time.

**Base pair**

The building blocks for DNA which are arranged in pairs, these can be either **A**denine, **C**ytosine, **G**uanine, and **T**hymine - i.e. A, C, G or a T.

**Common genetic variation**

Common genetic variation refers to all genetic variants across the genome in which the minor or rare allele occurs relatively frequently, that is, above 1%.

**Deoxyribonucleic acid (DNA)**

The helix molecule that encodes that the genome is made from.

**Dominance**

Dominance refers to the phenomenon whereby the effect of one allele masks the expression of another allele of a single gene.

**Dynastic effects**

Dynastic effects refer to the direct effects of parents’ phenotypes on their offspring. An example of this in the education context would be highly educated parents creating a nourishing learning environment for their children via buying books and helping their children learn to read. These effects are sometimes referred to as ‘genetic nurture’ (Kong et al., [2018](https://bera-journals.onlinelibrary.wiley.com/doi/full/10.1002/berj.3466#berj3466-bib-0018)).

**Epistasis**

Epistasis refers to the phenomenon whereby the phenotype of one gene can be modified by others. An epistatic gene refers to the gene whose phenotype is expressed.

**Gene**

A stretch of DNA formed by a distinct sequence of nucleotides constituting a section of a chromosome. Coding regions (exons) of the gene encode protein and are interspersed with non-coding regions (introns). This is distinct from a genetic variant, which can occur in a gene (intragenic) or outside a gene (intergenic).

**Gene-environment correlation**

Gene-environment correlations occur when SNPs occur at different frequencies in different environments.

**Genome**

The helix of DNA contains approximately three billion base pairs, of which around 7 million common genetic variants within the population.

**Genetics/genomics**

Research using genetic data.

**Genetic relatedness matrix**

Identical twins share all their germline genome and have a genetic relatedness of one. Siblings share half their genetic code and have a genetic relatedness of 0.5. If two unrelated individuals drawn at random from the population are more genetically similar than you would expect by chance, they will have a genetic relatedness of greater than zero. If they are less alike than you would expect by chance, they will have a genetic relatedness of less than zero. The genetic relatedness matrix stores the genetic relatedness between every pair of individuals in a sample. It can be used to estimate heritability using GCTA (see below).

**Genome-wide association study (GWAS)**

A genome-wide association study (GWAS) tests the associations of hundreds of thousands of genetic variants and an outcome (the phenotype). Due to the number of associations being tested simultaneously, and therefore issues of multiple hypothesis testing, strict P-value thresholds (conventionally 5 × 10−8) are used to account for multiple testing. The combination of these strict P-value thresholds and small SNP effect sizes means that GWASs require very large samples. GWASs are typically separated into two parts: an analysis is first performed in a discovery cohort to identify nominally genome-wide significant SNPs; and second performed in an independent validation cohort to validate these SNPs.

**Genome-wide Complex Trait Analysis (GCTA)**

Genome-wide complex trait analysis (GCTA) is a statistical programme that uses a genomic-relatedness-based generalised restricted maximum likelihood (GREML) approach to estimate the proportion of variance in a phenotype that can be statistically accounted for by all common SNPs. GCTA compares the genetic similarity between unrelated individuals and compares it to their similarity on phenotypic traits; where pairs of unrelated individuals are genetically and phenotypically similar, this provides evidence that phenotypic variation can be explained by genotypic variation. GCTA studies typically require sample sizes in the many thousands.

**Haplotypes**

Haplotypes are a specific sequence of alleles that are inherited together from a parent, leading to conserved sequences across generations.

**Hardy–Weinberg equilibrium**

Hardy–Weinberg equilibrium refers to the principle that genetic variation across a population will remain constant (in equilibrium) over generations in the absence of external disruptive or evolutionary factors. External factors that may disrupt Hardy–Weinberg equilibrium include non-random mating, mutations, genetic drift and natural selection.

**Heritability**

Heritability is the proportion of total phenotypic variance in a population that can be explained by genetic variance, and therefore ranges from zero (no phenotypic variance explained) to one (all phenotypic variance explained). Broad-sense heritability (H2) is defined as the total proportion of variance in a trait that is explained by all genetic variation, inclusive of additive genetic variance, dominance and epistasis (gene–gene interactions). Narrow-sense heritability (h2) is the proportion of total variance in a trait that is explained by additive genetic variance. SNP heritability
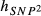
 is a measure of narrow-sense heritability calculated from a given set of genetic variants (SNPs). Heritability is a population rather than an individual parameter, and is specific to both the population and the environment under analysis.

**Heterozygosity**

Heterozygosity refers to the occurrence of two different alleles at a specific genetic locus.

**Homozygosity**

Homozygosity refers to the occurrence of two of the same alleles at a specific genetic locus.

**Identity by descent**

Where a segment of the genome shared by multiple people is due to inheritance from a common ancestor.

**Linkage disequilibrium**

Linkage disequilibrium refers to the combination of alleles at two or more loci occurring more frequently than would be expected by chance. This typically occurs for variants in close proximity in the genome. This is in violation of Mendel's second law of inheritance, which states that the identity of an allele should provide no information about alleles at other points in the genome.

**Meiosis**

Meiosis is the process by which a cell divides twice, creating four cells which each contain half a set of chromosomes as the original cell. These four cells are an organisms sex cells, making meiosis a (necessary) characteristic of organisms that reproduce sexually.

**Mendelian randomization**

The use of genetic variation as a natural experiment to learn about causal mechanisms.

**Non-inherited genetic variants**

At conception offspring inherit 50% of their mother’s genome, and 50% of their father’s. The half of their parents’ genomes that they do not inherit is called the non-inherited genome. It is impossible for the non-inherited genome to have biological effects on the children.

**Parent offspring trio**

A dataset of mother, father and offspring with both phenotypic data and genotypic data on each member of the family. This can allow both the inherited and non-inherited genetic variants to be identified.

**Phenotype/phenotypic trait**

A phenotype is the trait or characteristic of interest, for example: educational attainment, cognition and socioeconomic position are all phenotypes.

**Pleiotropy**

When a SNP is associated with multiple traits. This can vertical pleiotropy, in which a SNP affects a trait, which subsequently affects the outcome of interest (e.g. SNPs>educational attainment>smoking), or horizontal pleiotropy, in which a SNP has direct effects both a trait and an outcome of interest via other pathways (e.g. SNP>cognition>smoking and SNP>educational attainment).

**Polygenic trait**

Polygenic trait is the term used to refer to phenotypic traits that are influenced by many SNPs, the majority of which can only explain a very small proportion of variance in a trait. Most human behavioural traits that are influenced by our DNA are polygenic, being influenced to a small degree by hundreds or thousands of SNPs.

**Polygenic score**

A polygenic score (PGS, sometimes referred to as a polygenic risk score) is a summed score of the number of alleles associated with a phenotypic trait. These scores are often weighted by the genetic variant's effect size on the phenotype as estimated from a published GWAS. Polygenic scores can use genetic variants that were associated with the phenotype at different P-value thresholds ranging from genome-wide significance (p < 5×10^−8^) to liberal thresholds such as p < 0.5. Polygenic scores therefore indicate the summed influence that all genetic variants identified at a given level of GWAS significance have on a phenotypic trait. Because current GWASs are limited in the number of SNPs they can identify, polygenic scores based on these GWAS findings often omit many variants and therefore do not provide an estimate of the total genetic impact on a trait.

**Population stratification/structure**

Population stratification occurs when different subpopulations may have systematic differences in allele frequencies due to ancestral differences, such as non-random mating between subpopulations. These differences can occur because of geographical separation. The association of genetic variants and phenotypes can be confounded by population stratification. To control for population stratification, studies that estimate heritability use principal components analysis (PCA) applied to the genome-wide SNP data to infer population structure, then include the resultant principal components as covariates in analysis to account for population-specific variations in allele distributions.

**Reverse causation**

Reserve causation can occur when the outcome is caused by the exposure. For example, an analysis of short sightedness as an exposure and educational attainment as an outcome, would find a positive association between short-sightedness and educational attainment. However, this is likely because some aspect of the educational process affects myopia.

**Single nucleotide polymorphism (SNP)**

A single nucleotide polymorphism (SNP) is a genetic variant of a single base pair at a specific position in the genome.
